# The spatiotemporal distribution of Mesozoic dinosaur diversity

**DOI:** 10.1098/rsbl.2024.0443

**Published:** 2024-12-11

**Authors:** Philip D. Mannion

**Affiliations:** ^1^ Department of Earth Sciences, University College London, London WC1E 6BT, UK

**Keywords:** common cause hypothesis, Dinosauria, diversity, extinction, latitudinal diversity gradient, Mesozoic

## Abstract

Much of our view on Mesozoic dinosaur diversity is obscured by biases in the fossil record. In particular, spatiotemporal sampling heterogeneity affects identification of the timing and geographical location of radiations, the recognition of the latitudinal diversity gradient, as well as interpretation of purported extinctions, faunal turnovers and their drivers, including the Early Jurassic Jenkyns Event and across the Jurassic/Cretaceous boundary. The current distribution of sampling means it is impossible to robustly determine whether these ‘events’ were globally synchronous and geologically instantaneous or spatiotemporally staggered. Accounting for sampling heterogeneity is also paramount to reconciling notable differences in results based on sampling-standardized dinosaur species richness versus reconstructions of diversification rates, particularly with regards to the lead-up to the Cretaceous/Paleogene mass extinction. Incorporation of a greater proportion of stratigraphically well-resolved dinosaurs into analyses is also imperative and must include the substantial Mesozoic radiation of birds. Given the relative rarity of temporally successive, well-sampled spatial windows, it remains possible that dinosaur species richness and diversification rate showed little change after the clade’s initial radiation until the Cretaceous/Paleogene boundary. However, better understanding of underlying sampling, combined with a holistic approach to reconstructing dinosaur diversity and diversification, is an important step in testing this hypothesis.

## 1. Introduction

**Figure 1.**
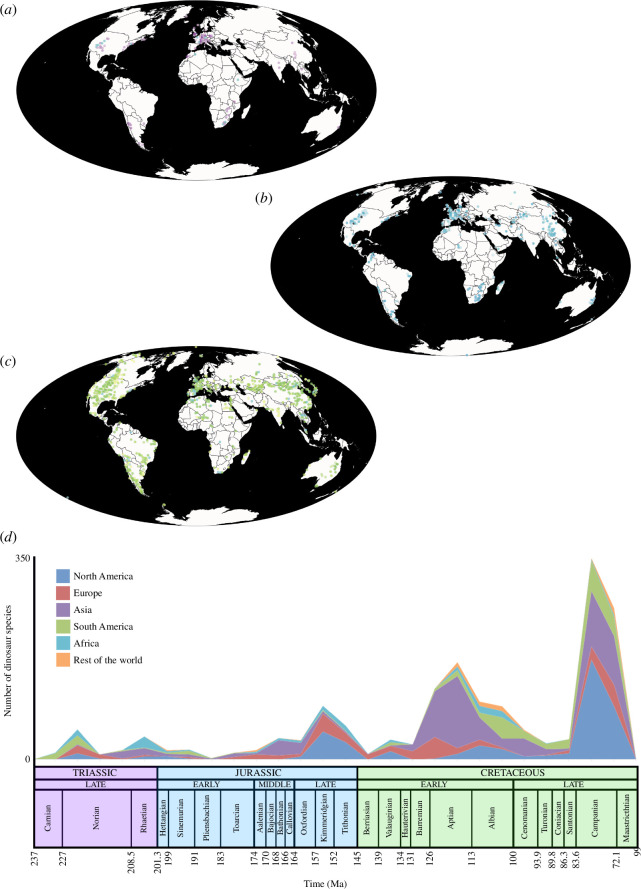
The spatiotemporal distribution of Mesozoic dinosaurs, including birds. (*a*) Spatial distribution of Late Triassic occurrences. (*b*) Spatial distribution of Jurassic occurrences. (*c*) Spatial distribution of Cretaceous occurrences. (*d*) Observed dinosaur species richness through the Mesozoic, showing contributions from each palaeocontinental region. Data in (*a*–*c*) plotted on a present-day Mollweide map projection using *The Paleobiology Database* navigator (https://paleobiodb.org/navigator/). Data in (*d*) plotted at the midpoint of each stratigraphic stage based on data in *The Paleobiology Database* as of 31 July 2024.

Dinosaurs first appear in the fossil record in the Middle or Late Triassic [1–4], approximately 230 million years ago (Ma). They diversified and dispersed to become a cosmopolitan radiation, dominating Mesozoic terrestrial ecosystems (e.g. [5,6]; figure 1*a*–*c*). Dinosaurs also survived two mass extinction events [1,2,7] and today represent one of the most speciose clades of terrestrial vertebrates [8]. Understanding how, when and why dinosaurs diversified remains a central macroevolutionary question, as does the nature of the extinction of all non-neornithine lineages at the end of the Mesozoic, 66 Ma [5–7].

A key component of addressing this centres on understanding how dinosaur diversity fluctuated through the Mesozoic. Diversity can be defined in numerous ways, but this contribution focuses on taxonomic rather than ecomorphological diversity, i.e. species richness. Many dinosaur studies focus on genus rather than species diversity; however, given that approximately 99% of dinosaur genera are monospecific [9], the unit of species is used herein for consistency throughout, even when the original study estimated diversity at the genus level. Globally, the number of species at any time is dependent on diversification rate, i.e. the net balance of origination and extinction, with dispersal also an important factor at regional scales. As such, diversification rate is an additional important measure when discussing dinosaur species richness.

Several authors have attempted to quantitatively estimate how many dinosaur species were alive during the Mesozoic, using a range of methods, including coverage and richness extrapolators. These estimates range from 900 to 3400 species in total during this 160 million year time interval [10–13]. In contrast, based on a species–area relationship, Le Loeuff [14] estimated that 628−1078 non-avialan dinosaur species were alive solely in the late Maastrichtian, which, if extrapolated, would imply an order of magnitude higher count for Mesozoic diversity compared with other estimates [5]. Based on data in *The Paleobiology Database* (https://paleobiodb.org/), approximately 1550 dinosaur species are currently recognized as valid for the entirety of the Mesozoic (figure 1*d*), of which approximately 225 species are birds. Using the more conservative estimate range, this would mean that between 45% and 100% of Mesozoic dinosaur species have already been discovered (with potentially a large number of currently recognized species representing *nomina dubia*), whereas extrapolations based on the Le Loeuff [14] estimate suggest that less than 10% of species have been discovered. This dichotomy in estimates provides a useful platform for considering how Mesozoic dinosaur diversity, and our sampling of it, varies through time and space.

## 2. Reconstructing patterns in dinosaur diversity and diversification

In addition to trying to calculate how many species of dinosaurs were alive during the Mesozoic, a number of studies have sought to determine how the group’s diversity varied through time. The earliest analyses reconstructed this by counting the number of species in each time interval [10,15–17]. However, reading the fossil record at face value had long been recognized as problematic, with apparent peaks and troughs in diversity through time potentially merely artefacts of heterogenous sampling [18] (see below).

### (a) The impact of sampling

Unsurprisingly, the discovery and description of new species have an effect on reconstructions of dinosaur diversity and diversification [6,19,20]. This includes the extension of clades backwards and forwards in time, as well as in geographic spread (e.g. [21–23]), but increases in sampling more broadly have a notable impact on overall patterns, particularly for spatiotemporal windows that have been historically undersampled [20,24–26].

**Figure 2.**
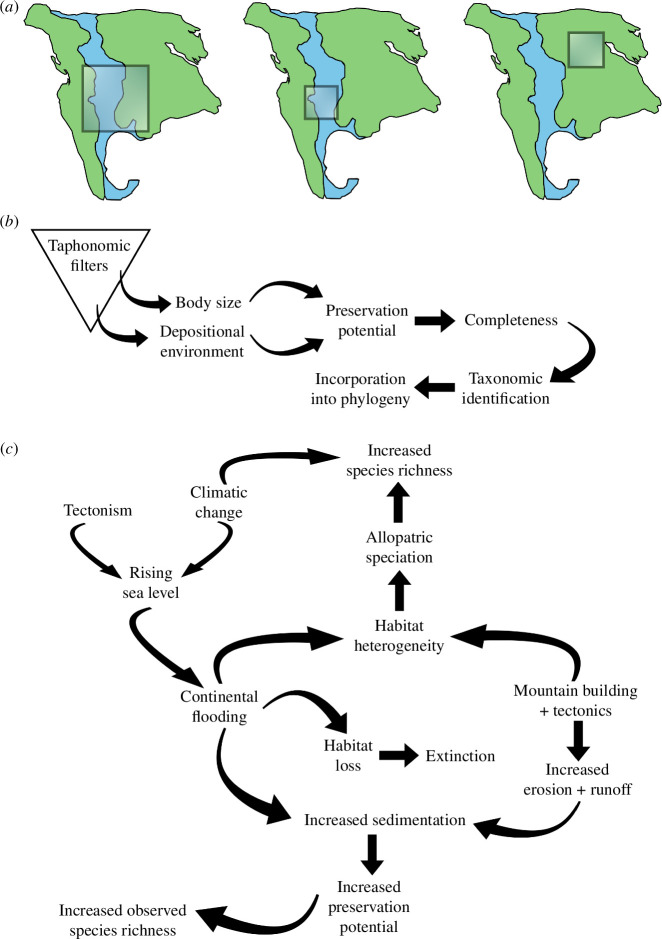
Impact of sampling on dinosaur diversity. (*a*) Schematic of the spatial sampling filter that affects consistency of sampling effort, with differing sampling coverage (i.e. size, location and environment) dictating observed diversity. (*b*) Taphonomic filter that affects preservation and recognition of species and their inclusion in analyses of dinosaur diversity. (*c*) Terrestrial common cause scenarios that affect both genuine and observed species richness.

A suite of underlying sampling biases influences the available fossil record [27] (figure 2). Of these, perhaps the most obvious and pervasive pertains to the spatial distribution of sampling, both in terms of the availability and accessibility of suitable sedimentary outcrop [9,12], but also with regards to collection effort [28] (figures 1 and 2*a*), which shows a clear socioeconomic bias towards high- and upper-middle-income countries [26]. The spatial coverage of the sampling window within any time interval species will therefore dictate the diversity that can be observed (figure 2*a*). Consequently, studies of dinosaur diversity through time that have typically relied on global estimates are problematic, given that the ‘global’ sample in each time interval varies substantially in terms of spatial extent [29,30]. These problems also encompass issues pertaining to data absence, i.e. whether a species was genuinely absent from a spatiotemporal window or if it is merely not sampled (=sampling failure), which affects estimates of species richness, origination and extinction [31].

A number of studies have demonstrated statistically significant correlations between dinosaur subclades and the type of depositional environment from which specimens are most frequently recovered [32–37]. As such, if our spatiotemporal sampling is not environmentally homogeneous, then reconstructions of diversity and diversification patterns might also be affected by this, with some clades unsampled (figure 2*b*). As an additional complication, the environment of deposition also has a taphonomic effect, with certain settings more suitable for preservation, and these are not necessarily consistent across clades, especially given substantial disparity in body size and skeletal fragility [38–41].

The completeness of skeletons also affects our interpretation of dinosaur diversity and diversification patterns [38,42,43]. Body size plays a role with regards to completeness (figure 2*b*), with a bias against the preservation of smaller sized taxa (approx. < 70 kg), except those discovered within Lagerstätten deposits [5,38,40,44,45]. Among larger bodied dinosaurs, there is less of a clear correlation between size and completeness [25,46]. Low levels of completeness can result in unrecognized diversity because of an inability to identify new species (figure 2*b*). Conversely, it can also lead to an inflation in diversity through the erection of synonymous species based on non-overlapping elements [43,47]. Furthermore, species known from highly incomplete specimens are less likely to be incorporated in phylogenetic analyses (figure 2*b*), and their placement is often highly labile when they are included. As such, they are often excluded from phylogeny-based diversification analyses.

The choice of time bin duration appears to have little effect when evaluating large-scale diversity dynamics throughout the Mesozoic (e.g. [48]), but its impact is more apparent at finer temporal scales [49,50], such as when attempting to decipher patterns in the lead-up to the end-Cretaceous extinction (see below). One particular problem is that non-contemporaneous stratigraphic units (and by extension the species within them) are often lumped together, giving misleading time-averaged notions of species richness that can also have notable effects on reconstructions of spatial diversity patterns, including identification of endemism [31,51–53]. Cashmore *et al.* [25] re-evaluated temporal patterns in fossil record completeness of sauropodomorph dinosaurs, testing for the effect of 10 years of new data on the results of an earlier study [43]. They demonstrated that the most substantial change resulted from the revision of stratigraphic ages of fossil occurrences, which potentially has important ramifications for diversity analyses given that equivalent corrections have yet to be made for datasets utilized for theropods and ornithischians.

### (b) Analytical solutions to sampling heterogeneity?

Although a small number of authors have continued to take a literal reading of the dinosaur fossil record [54–56], most subsequent studies have attempted to address the critical issue of sampling heterogeneity. This includes the incorporation of phylogenetic relationships to capture unsampled ghost lineages [9,57–63], the calculation of residuals from a modelled relationship between diversity and a proxy for sampling [9,28,38,48,62,64,65] and application of a richness extrapolator [13]. However, all of these approaches are problematic in that they do not appear to resolve underlying sampling issues, especially those pertaining to spatial and temporal heterogeneities [66–71].

This has left two primary approaches that authors have used to reconstruct dinosaur diversity: sampling standardization and estimates of diversification rates. Fastovsky *et al.* [72] were the first to apply sampling standardization to estimate dinosaur species richness, demonstrating that some of the face-value patterns described by earlier authors were sampling artefacts. Sampling-standardization approaches have since become a mainstay of attempts to reconstruct dinosaur diversity [7,20,29,49,62,73–77], with most analyses using some form of shareholder quorum subsampling (SQS), also known as coverage-based rarefaction [78,79]. However, estimating species richness through time in this way does not allow us to determine whether fluctuations are a result of changes in origination or extinction rate, or both. A second subset of analyses has therefore focused on reconstructing diversification rates, either based on fossil occurrences, using SQS [76] or PyRate [63,80], which incorporates a measure of sampling rate, or via analyses of phylogenetic trees [61,63,81–86].

As outlined in the preceding section, there is a substantial problem with estimating ‘global’ diversity. This issue was identified in earlier studies of the marine realm [87,88] but was not explicitly considered in initial analyses of dinosaur diversity. Whereas some studies attempted to ameliorate this issue through the reconstruction of regional (=continental) diversity curves [7,20,28,75,76,89], subsequent analyses have incorporated a spatial component to the subsampling approach [29,77]. Neither of these considerations has yet to be fully factored into reconstructions of dinosaur diversification rates [80,83,85,86].

A remaining problem that neither type of analysis can ameliorate is the issue of genuine versus pseudo-absence. This has been recently addressed through the application of ecological niche modelling, in which dinosaur habitat suitability is projected into space, shedding light on whether unsampled regions could have hosted dinosaur species [31,90,91]. However, although this can aid in determining whether an apparent diversity trough represents a genuinely depauperate spatiotemporal window, it cannot necessarily be used as a proxy for species richness [6].

## 3. Mesozoic dinosaur diversity and diversification

Whereas debate often centres on whether or not extinction events are genuine, one often neglected impact of spatiotemporal sampling heterogeneity is the possible misidentification of radiations. Well-sampled spatiotemporal intervals with high taxonomic diversity of a particular clade are often regarded as capturing genuine radiations, with such an interpretation regularly supported quantitatively by biogeographical analysis. Below, several purported radiations and extinctions during the Mesozoic are discussed in this light. Given the issues raised above, including the relative rarity of temporally successive, well-sampled spatial windows (figure 1*d*), it remains possible that Mesozoic dinosaur diversity and diversification rate showed little in the way of change after the clade’s initial radiation (see also [5,29,75,77]).

### (a) Did dinosaurs originate in the early Late Triassic of southern Gondwana?

**Figure 3.**
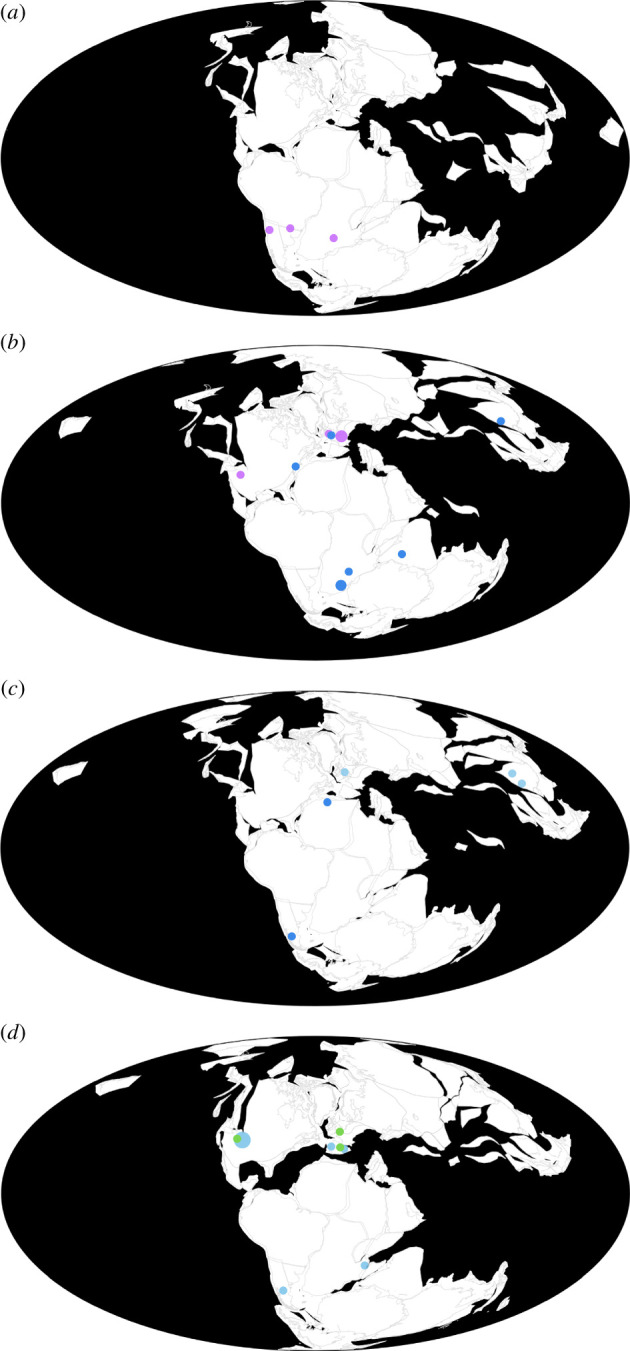
Palaeogeographic reconstructions of Mesozoic intervals showing spatial distribution of well-sampled dinosaur body fossil-bearing deposits. (*a*) Carnian (purple circles; plate reconstruction = 232 Ma); (*b*) latest Triassic (purple circles) to earliest Jurassic (dark blue circles; plate reconstruction = 200 Ma); (*c*) Toarcian (dark blue circles) to early Middle Jurassic (light blue circles; plate reconstruction = 179 Ma); (*d*) Kimmeridgian–Tithonian (light blue circles) to earliest Cretaceous (green circles; plate reconstruction = 147 Ma).

The stratigraphically earliest known unequivocal dinosaurs come from the upper Carnian (lower Upper Triassic) Ischigualasto and Santa Maria formations of Argentina and Brazil, respectively [3,92], as well as the Carnian Pebbly Arkose Formation of Zimbabwe [93] (figure 3*a*). As a result of the high taxonomic and morphological diversity of dinosaurs in these formations, alongside their absence from Laurasia until the Norian, nearly all studies regard southern Gondwana as the likely centre of origin for the clade [94]. However, this does not consider the possibility that unsampled contemporaneous regions (figure 1) might also have harboured dinosaurs. Perhaps more importantly, it does not take into account that dinosaurs presumably evolved prior to the late Carnian, as evidenced by putative earlier members (e.g. [4]), alternative phylogenetic topological arrangements (e.g. [95,96]) and divergence time estimates [97], and thus potentially originated elsewhere. These considerations also potentially complicate interpretations of the Carnian Pluvial Event as a driver of early dinosaur diversification [98,99].

### (b) The Triassic/Jurassic boundary

Although recognized as the one of the ‘Big 5’ Phanerozoic mass extinctions, dinosaurs appear to have passed through the Triassic/Jurassic (T/J) boundary relatively unscathed. There is little evidence for substantial losses in dinosaur diversity or a reduction in diversification rate across the boundary, with some studies indicating that the extinction event paved the way for the clade’s subsequent radiation [1,2]. Nevertheless, most analyses have been based on a ‘global’ appraisal of the T/J transition (e.g. [9,63,81]). Sampling within the time intervals either side of the boundary is spatially disparate (e.g. [20,28,75]; figures 1*d* and 3*b*), meaning that it is essentially impossible to confidently determine dinosaur diversity dynamics across the T/J boundary. Nevertheless, the survival of most dinosaur lineages across the T/J boundary suggests that the clade was less affected by the extinction event than other taxonomic groups, which in itself remains an important unanswered conundrum.

### (c) The Jenkyns Event and Middle Jurassic dinosaur radiation

A number of studies have discussed dinosaur diversity patterns during the late Early to Middle Jurassic. These contributions have highlighted a faunal turnover tied to heightened activity of the Karoo–Ferrar Large Igneous Province in the early Toarcian (the Jenkyns Event), followed by the subsequent radiation of multiple lineages [100–102]. However, although there is a clear difference in taxonomic composition between late Early Jurassic and Middle Jurassic dinosaurs (e.g. from ‘basal’ sauropodomorph- to eusauropod-dominated faunas), there are also substantial spatiotemporal sampling gaps during this transition (figures 1*d* and 3*c*), and very few dinosaur-bearing deposits can be unequivocally dated to the early Middle Jurassic [13,28,62]. As such, it is possible that the apparent faunal turnover was neither globally synchronous nor geologically rapid, and that the rise of ‘derived’ dinosaur lineages that went onto dominate ecosystems in the Middle–Late Jurassic onwards was spatiotemporally staggered.

### (d) Is diversity in the Late Jurassic unusually high?

The Late Jurassic is often highlighted as the heyday of sauropod dinosaur diversity (e.g. [60]). Much of this derives from the high species richness recorded in the Upper Jurassic Morrison Formation of the USA (figures 1*d* and 3*d*), particularly with regards to diplodocids, and which is frequently cited as evidence for a North American origin of this clade (e.g. [103]). However, this formation is essentially a form of Lagerstätte, with a long history of dinosaur fossil collection stemming back to the late nineteenth century. Interpreting its high diversity at face value is similar to regarding the rich avialan fossil record preserved in the late Early Cretaceous Jehol Biota (e.g. [104]) as capturing the actual diversification of birds. In both cases, these represent exceptional windows into diversity within a particular spatiotemporal interval; however, there is no *a priori* reason to assume that species richness was not comparable in other spatiotemporal windows and thus that these radiations could not have happened earlier and/or elsewhere. This reasoning is borne out by analysis of alpha diversity, which shows broadly consistent upper levels of local richness for dinosaurs throughout much of the Mesozoic [105].

Furthermore, new species are still being discovered in the Morrison Formation (e.g. [106]), as well as from other stratigraphic units and localities generally regarded as well sampled for dinosaurs, such as the latest Cretaceous Dinosaur Park and Hell Creek formations of North America and the contemporaneous Nemegt Formation of Mongolia (e.g. [44,107]). Small-bodied dinosaur species, including avialans, are also typically exceedingly rare in many formations otherwise thought to be well sampled, meaning that substantial subsets of these ecosystems are missing (e.g. [5,44,45]). When even collector curves from stratigraphic units with historically long fieldwork programmes and extensive fossil records are yet to approach an asymptote (e.g. [107]), this indicates that the diversity of other formations is likely to be heavily undersampled.

### (e) Was there a mass extinction at the Jurassic/Cretaceous boundary?

Originally regarded as a mass extinction event, dinosaur diversity patterns across the Jurassic/Cretaceous (J/K) boundary remain uncertain [6,76,108]. This interval perhaps best exemplifies many of the issues surrounding sampling bias and heterogeneity outlined above, as well as how our ideas of dinosaur diversity can change.

As an example of the problems associated with deciphering this interval, diplodocids were initially thought to have gone extinct at the J/K boundary [60], following their apparent Late Jurassic boom. The discovery of Cretaceous Gondwanan representatives showed that this was incorrect [109], but their continued absence from the Cretaceous Laurasian record suggests that they might have become extirpated at the J/K boundary in the Northern Hemisphere. Until recently, the dearth of pre-Barremian terrestrial deposits in North America meant that it was impossible to determine whether the Morrison Formation diplodocids died out at the J/K boundary or at some point in the subsequent 20 million years. However, the revised chronostratigraphic framework for the unconformably overlying Yellow Cat Member of the Cedar Mountain Formation in Utah (figure 3*d*) dates this stratigraphic unit to the upper Berriasian–Valanginian, substantially closing this temporal gap [110]. Given the preservation of other sauropod clades in the Yellow Cat Member, this suggests that the absence of diplodocids is genuine and provides some support that the demise of North American representatives of the clade occurred at or close to the J/K boundary [110].

As demonstrated in this one example, new discoveries, revised stratigraphy and consideration of regional rather than ‘global’ diversity can all contribute to our interpretation of this one time interval. Currently, our understanding of dinosaur diversity across the J/K interval hinges primarily on the European record (figures 1*d* and 3*d*), which provides evidence for a prolonged period of turnover rather than a discrete extinction event [76]. It remains to be seen whether changes in dinosaur diversity were global and/or synchronous, which also makes it difficult to hypothesize which of the contemporaneous environmental perturbations might have been responsible [108].

### (f) Turnover and diversification in the Late Cretaceous

Evidence for a faunal turnover in dinosaur communities during the Cenomanian–Turonian (early Late Cretaceous) interval (figure 1*d*) has been presented by several authors (e.g. [111]), tied to environmental perturbations resulting from Oceanic Anoxic Event 2 and the Cretaceous Thermal Maximum (see [89]) and potentially linked to the angiosperm radiation (see [6,112]). Our understanding of this interval is affected by similar problems as the Early–Middle Jurassic interval (see above), including poor chronostratigraphic constraint in some regions. This is exacerbated by a proportional decrease in terrestrial sedimentary outcrop as a result of the Turonian sea-level highstand, with limited global sampling until the Campanian [62,89]. Taking sampling heterogeneity into consideration, there is currently no evidence for a globally synchronous or geologically instantaneous faunal turnover during the Cenomanian–Turonian interval [28,48,89,113], with little change in reconstructed diversification rates at this time either [80,81]. Increasing chronostratigraphic resolution of relevant successions [110,114,115] should allow this to be more robustly tested in the future.

Gates *et al.* [116] suggested that orogenic events led to dinosaur diversification in the Late Cretaceous of North America, and mountain-building tectonic events have been proposed as an important mechanism in driving mammalian diversity dynamics in both the Mesozoic and Cenozoic (e.g. [117]). Although basin- and larger-scale tectonics almost certainly would have impacted dinosaur diversity, e.g. through stimulating allopatric speciation and generating habitat heterogeneity [116–118], it has a dual role in sampling, whereby increased sedimentation and run-off would have led to heightened fossil preservation potential and thus greater observed diversity [31,53] (figure 2*c*). This ‘common cause’ scenario has been well studied with regards to the effects of sea level (figure 2*c*), including for Mesozoic dinosaurs [48,76], but is yet to be evaluated at a comparable level for tectonism.

### (g) Sudden versus gradual extinction in the latest Cretaceous?

Despite a 160 million year record, much of the published literature on dinosaur diversity has focused on the last 10 million years of the Mesozoic. This has centred on whether or not dinosaurs were in long-term decline prior to the demise of all non-neornithine members of the group, as well as the cause(s) of their extinction (see reviews in [6,7,55,119,120]). Studies that have taken the fossil record at face value have typically supported a gradual extinction [10,15,54,56,119] (figure 1*d*), whereas most sampling-standardized approaches indicate little evidence for diversity decline prior to the Cretaceous/Paleogene (K/Pg) boundary, implying that the extinction was geologically instantaneous [7,72]. In contrast, reconstructions of dinosaur diversification rate typically support longer term decline [80–82] (though see [83,85,86]).

This discussion has been dominated by studies of the well-sampled North American record, for which detailed work on local sections has shaped our views on dinosaur diversity dynamics in the lead-up to the end-Cretaceous extinction (e.g. [121–124]). These rich study systems provide a template for how a detailed understanding of regional diversity, based on high-quality, well-constrained datasets, can be scaled to infer global dynamics. Data from other regions can potentially be brought to bear on this topic, with increased sampling and stratigraphic resolution in the last decade, particularly for Asia, Europe and South America (e.g. [56,125,126]), which will enable us to eventually test whether the North American pattern represents a global signal. However, analyses of these regions also need to account for spatiotemporal sampling heterogeneity.

Uncertainty in the temporal nature of the extinction has also led to a long-standing debate with regard to the kill mechanism. Whereas the bolide impact that struck the Earth at the K/Pg boundary is coincident with the demise of the non-neornithine dinosaurs and explains their sudden extinction (e.g. [90]), a more gradual decline has typically been explained via the impact of Deccan volcanism and other environmental perturbations (e.g. [80]). A clearer understanding of dinosaur diversity dynamics outside of North America might shed light on this issue.

One problem with nearly all of these studies is that they typically evaluate a paraphyletic Dinosauria that excludes birds. Yet, approximately 15% of currently recognized Mesozoic dinosaur species are birds, and we know that avialan dinosaurs represent a substantial Mesozoic radiation that is almost certainly undersampled as a result of taphonomic filters (see above). This includes an increase in diversification rate during the Late Cretaceous that corresponds to the initial radiation of crown birds [84], the first appearance of neornithine birds in the latest Cretaceous fossil record [127,128] and a diverse latest Cretaceous avifauna [129]. The exclusion of birds from dinosaur diversity analyses is equivalent to omitting ornithopods or titanosauriforms, groups that are each similarly speciose to Mesozoic birds, and which would artificially result in lowered reconstructed diversity in Cretaceous ornithischians and the apparent extinction of sauropods in the early Late Cretaceous, respectively. Although the different preservation potential of bird skeletons compared with most other dinosaurs is a potential reason to evaluate avialan diversity separately (e.g. [38,39]), many non-avialan paravian theropod dinosaurs have similarly delicate bones yet are evaluated alongside their larger-bodied dinosaurian relatives.

An important consideration is that measures of diversity and diversification rate are not interchangeable: it is possible that species richness remained high while diversification rate showed overall decline [82]. Finally, if there was a decline in dinosaur diversity or diversification rate in the latest Cretaceous, this does not necessarily mean that the group was headed for extinction [7]. Previous time intervals also show evidence for comparable stage-to-stage diversity declines [9,13,28]; yet, dinosaurs not only did not go extinct at these times, but experienced subsequent diversifications.

## 4. The latitudinal diversity gradient of Mesozoic dinosaurs

Whereas most studies to consider dinosaur diversity have evaluated temporal patterns, how the distribution of this species richness varied spatially is also important. This is arguably of paramount importance given that our understanding of diversity dynamics through time is dependent on spatial sampling heterogeneity. Biodiversity through time is entwined with biodiversity through space and the two must be considered together [30]. This is perhaps best exemplified by the latitudinal diversity gradient (LDG).

In the present day, species richness is greatest in the tropics and declines towards the poles, representing a first-order macroecological pattern; yet, attempts to understand the LDG are precluded by covariation with latitude of the various proposed drivers [130]. In contrast, many of these variables were decoupled in the Mesozoic [131]. The few studies to quantitatively evaluate how the distribution of dinosaur diversity varied with latitude have all recovered evidence for a flattened LDG, with a peak in diversity at temperate palaeolatitudes [50,73,91,132]. Although these studies accounted for latitudinal sampling heterogeneity within temporal windows, there are still problems pertaining to local versus ‘global’ sampling coverage [29], with no attempt to factor in longitudinal sampling heterogeneity. Nevertheless, alternative approaches to evaluating the dinosaur LDG, utilizing climate data and ecological niche modelling [91,132,133], reinforce the view that the distribution of dinosaur diversity did not conform to the present-day unimodal LDG, which likely formed only during the late Cenozoic [131]. Collectively, these studies also suggest that the diversity of sauropodomorphs was more latitudinally restricted than that of ornithischians and theropods [73,91,132,133], with these latter clades extending into polar regions (e.g. [134]).

## 5. Future directions

Returning to the question of how many dinosaurs were alive during the entirety of the Mesozoic, extrapolations based on Le Loeuff [14] appear to be more realistic than other published estimates given the high number of observed species despite pervasive spatiotemporal sampling failure. Although this contribution might seem overly pessimistic about our current understanding of Mesozoic dinosaur diversity, an increased awareness of the problems pertaining to sampling heterogeneity and recognition of the incomplete nature of the underlying data are important first steps. The last couple of decades have witnessed great strides in our approaches to addressing questions pertaining to dinosaur diversity and diversification, including increasingly refined and sophisticated methods.

Although the underlying datasets are richer and more readily available than ever, the quality and quantity of these data require refinement. Both occurrence-based and phylogenetic methods require stratigraphic information on species, and yet few analyses utilize updated stratigraphic data on dinosaurs [25,49]. Occurrence-based methods essentially include all Mesozoic non-avialan dinosaur species, but most phylogeny-based methods sample only approximately a third of this diversity (though see [82]), and most studies exclude birds regardless of approach. Improved chronostratigraphic resolution and incorporation of a greater proportion of dinosaur diversity, including birds, are necessary steps to elucidating diversity dynamics in this clade, as well as for enabling inter-model comparisons.

All studies of dinosaur diversity and diversification must consider both temporal and spatial sampling heterogeneities. Global reconstructions of diversity dynamics are problematic at best, and this affects both sampling standardization and diversification rate approaches. The development of sampling-standardized regional diversification rate estimates will be an important next step, allowing a fairer and more meaningful comparison with results emanating from sampling standardization-based analyses. Such approaches have recently been developed and applied to estimations of diversification rates in the marine fossil record [135,136], providing a starting point for their incorporation into analyses of dinosaur diversification. Similar spatiotemporal sampling problems also pervade ‘global’ reconstructions of time series of environmental variables [137], which are often used to test for correlations with dinosaur diversity dynamics. As such, it is also important to consider spatially appropriate environmental variables to provide meaningful tests of drivers of dinosaur diversity and diversification, whilst also taking into account the possibility of common cause effects (figure 2*c*).

Approaches such as ecological niche modelling (e.g. [31]) and occupancy modelling (e.g. [138]) can also be utilized to better understand whether absences of species from particular spatiotemporal windows are genuine or artefactual. Studies addressing diversity patterns within a time interval need to consider sampling heterogeneity latitudinally but also longitudinally. Biogeographical analyses need to factor in sampling heterogeneity too, and future studies should consider whether a sampling standardization approach is required both for occurrence-based and phylogenetic biogeographic methods.

Finally, there is no reason not to consider a more holistic approach to evaluating dinosaur diversity dynamics. Both species richness and diversification rate are important measures. Each of the approaches discussed herein have their problems, which might be overcome by bringing a range of methods to bear on this topic.

## Data Availability

This article has no additional data.
